# A Case of Septic Poisoning from Bed Sores

**Published:** 1888-11

**Authors:** F. A. Schmitt

**Affiliations:** Shulenburg, Texas


### A CASE OF SEPTIC POISONING FROM BED SORES.

BY F. A. SCHMITT, M. D., SHULENBURG, TEXAS.

[For DANIEL'S TEXAS MEDICAL JOURNAL.]

ON January 13th I was called in consultation to a case, the history of which, as communicated to me by the family physician, Dr. Fahnert, is as follows :

Mrs. S., aged 18, had been delivered of her first child during the month of December, 1887. She had symptoms of malarial fever a few weeks previous to her confinement. She was attended during delivery by a neighbor—a so-called midwife. Everything seems to have been natural, and on the tenth day she was up for the first time. On the twelfth day she was seized with a chill, followed by fever. Lochial discharge ceased from then on, and fever of an intermittent type continued two weeks, when swelling of right leg supervened, accompanied by high fever and pain.

My first visit was made five weeks after delivery, her condition then being: Temperature, 102; pulse, 110; white swelling (*phlegmasia dolens*) of right leg, and other extremities exceedingly painful to the touch; tongue, normal. Complained of pain in the region of sacrum, and I was told that redness preceding decubitus was there present; appetite good, and craving acidulated drink; excessive perspiration occasionally. Digital examination, which, on account of sensitiveness of lower extremities, was made with some difficulty, revealed vaginal tract and womb in normal condition.

Ordered nourishing diet, mineral (sulphuric) acid drink, and quinine—of which she had received large doses previously—in tonic doses. I furnished them an air cushion the next day, they living twelve miles in the country. For four days afterwards it was impossible for the family physician or myself to make the trip, on account of one of the severest cold spells I had ever experienced in Texas, but on the sixth day I managed to meet the attending physician again at the bedside.

Meanwhile, our patient had been comparatively well; her temperature had not augmented nor her pulse increased; appetite had been good, but to my great surprise she had not had the benefit and comfort of the furnished cushion, which, as I was told, had become defective while taking it thither, as I knew it to have been in good condition when delivered to messenger. I was sadly disappointed in this, as I had before explained to her people that her condition would perhaps necessitate a horizontal position for some time to come, it then being an utter impossibility to change the position in the least, on account of the excruciating pain every movement would produce, and that it was of the utmost importance to guard against bed sores by protecting projecting points. On arriving at home I at once had a second cushion ordered from New Orleans by telegraph. This was received by mail on the third day after wiring, and it was immediately sent out by special messenger.

My third visit was made nine days after my second, during which time her general condition had not changed for the worse, but she neither had the cushion under her—her attendants having tried to put it in position but had failed to place it so as to comfort her and it had again been removed.

We were now determined to place her on the cushion at all hazards but we soon became convinced that this could not be accomplished without the use of chloroform.

The glimpse we received of the sore on this occasion, sufficiently indicated that energetic antiseptics would be required to disinfect lesion, and as our saddle-bags were but scantily provided with these agents, I concluded to go home and return the next morning prepared with everything needed.

On turning her the next day while under the influence of chloroform, a most horrible sight met our eyes. A slough, weighing several ounces, dropped from out the seat of contusion laying bare a large part of the os sacrum and coccyx—the connection of these severed—and both bones in a necrotic condition.

After having thoroughly cleansed and disinfected the parts, we dressed the wound with carbolized absorbent cotton, placed her on the round air cushion in such a way as to bring the wounded surface within the hollow centre and ordered Tr. Fer. chlor. two drachms, Quinine sulph. one-half ounce, water six ounces—tea spoon full every four hours—and opiates to relieve pain—to be given. The wound to be dressed at least once a day.

During the night the first symptom of septicæmia in the form of a rigor set in, followed by high temperature—104—and profuse perspiration.

A third colleague, Dr. Grace, of Weimar, met us the next day in consultation, who assisted us in dressing the wound and prescribing for the doomed patient, and with whom I arranged alternate visits every second day. From then on rigor after rigor followed in rapid succession, large spots on back and shoulders became gangrenous, tympanites supervened, and the fifth day after the first dressing, ended a scene terrible to behold, and terminated a case as defiant to treatment as anything I had ever experienced.

